# Pelvic Abscess

**Published:** 1886-01

**Authors:** Henry T. Byford

**Affiliations:** 3100 Forest Avenue; Chicago


### REPORT OF A CASE OF PELVIC ABSCESS, WITH REMARKS UPON THE TREATMENT. *By* HENRY T. BYFORD, M. D., *Chicago.*

(Read before the Chicago Gynæcological Society, December 18th, 1885.)

Mrs. T., aged twenty-five years; married five years; German descent; of nervous temperament; small and slight in figure, but in good general health, consulted me, during the fall of the year 1884, for sterility and dysmenorrhœa. She had never menstruated without pain, but had otherwise enjoyed good health. An examination revealed a small uterus and cervix, with acute anteflexion and consequent apposition of the anterior and posterior uterine walls. Slippery elm tents, used about once in eight days, alternated with glycerine tampons, had for their effect a gradual relief of the dysmenorrhœa.

About the middle of the following February, I was called to her house to treat her for pelvic cellulitis, contracted a week before while returning home from a dance. The whole pelvic connective tissue seemed involved, and large tender lumps could be felt externally in the left iliac region.

Six weeks from the beginning of the attack, an abscess opened into the anterior wall of the rectum, about two inches from the external anal orifice. On account of the extreme debility of the patient, her horror of operative procedures, and the absence of any well marked fluctuation, all surgical interference with the suppurative process had been out of the question.

Attempts were then made at thorough antiseptic treatment, but neither elastic nor flexible metal tubes, although easily introduced into the abscess, could be tolerated there longer than a few hours at a time. Antiseptic irrigations, of carbolic and boracic acid solutions used four times a day, were then relied upon, but were inefficacious, for the pockets often discharged foul-smelling pus soon after the dressings.

In the meantime the pulse remained in the neighborhood of 120°, and the temperature fluctuated between 99° and 102° F.; attacks of acute suffering and septicæmic diarrhœa required opiates for their relief; the bacillus tuberculosis was discovered in the pus; yellow pigmentary deposits covered her face, and emaciation became extreme, her weight ranging between eighty-two and eighty-three and one-half pounds. Her courage began to fail, and finally, after the concurrent recommendation of the consultants, Drs. Wm. H. Byford, J. E. Owens, Geo. M. Chamberlin and Martin Matter, she consented to an operation.

Accordingly on the 6th of June Dr. Wm. H. Byford operated according to his usual method in such cases. After etherization, he forcibly dilated the sphincter of the anus, tore open the fistulous track with the finger, and then enlarged the opening in the same manner, in the direction of the lowest part of the cavity, until it readily admitted two fingers. I then made a digital examination, and found the abscess to extend across the pelvis, behind the uterus and broad ligaments, to a point above the level of the fundus uteri on the left side, and to be filled with bands and projecting masses of granulation-tissue of about the consistency of freshly coagulated blood. Previous treatment, except to diminish and control the septicæmia, had evidently been a complete failure. All of this medullary tissue was then scooped out with the finger and the cavity thoroughly cleansed with a two and a half per cent. solution of carbolic acid.

The highest temperature after the operation was 99° F., on the day following. Perfect drainage had been secured, for at the time of each dressing no pus was found inside of the abscess.

June 12th. Abscess-opening still an inch in diameter, without any apparent induration about its edges. Temperature normal. Patient allowed to get up. Expressed herself as feeling perfectly well.

June 16th. Took a buggy ride with benefit. Weight eighty-four pounds.

June 23d. A little pus found in the abscess at each dressing. Sphincter ani firm and contracted, causing some trouble by retaining pus, and interfering with irrigation. Attempted forcible dilatation with the aid of cocaine hydrochlorate, but failed to secure sufficient anæsthesia. Abscess-opening contracting.

June 25th. A. M., temperature 99 2-5° F.; P. M., 99 3–10.

June 28th. A. M., temperature 98 4–5. Not much discharge. Pain in iliac region and breasts, such as she formerly felt before menstruating. Abscess-opening almost closed by the uterus being drawn back over it. Would not consent to another operation.

June 29. An increase of discharge.

June 30. Dilated the abscess-opening with a large sponge tent, and washed it out thoroughly.

July 1. Temperature normal. Pains gone. Weight eighty-eight pounds.

She was now allowed to visit her relatives in Chesterton,

Indiana, where she came under the care of Dr. D. D. Marr. He continued the antiseptic irrigations, and used sponge tents as often as the opening contracted. He reported to me as follows:

"July 8th. Patient has gained two and a half pounds "in weight. Pulse 115.

"July 16. Gain of half a pound. Less discharge. A sup-"pository of iodoform is left in abscess after each dressing.

"July 28th. Patient much improved. Rode twenty miles "a few days ago in a buggy. Heavy deposits of phosphates in "the urine. A pocket of pus is giving trouble by filling and "discharging. Iodoform insufflations commenced.

"Aug. 12th. Abscess was closed up for a few days. This "was accompanied by night-sweats and pains, and was soon "followed by a copious discharge of fetid pus."

After consulting together we decided to allow a solid piece of sulphate of copper to dissolve in the abscess-cavity.

Aug. 20th. The doctor writes: "Owing to an epidemic in "a little village near here I have been very busy and put off "reporting Mrs. T's condition. I placed a piece of sulphate "of copper, weighing a dram and a half, in the abscess (well "up), which had a most wonderful and I hope happy effect. " * * * The opening of the abscess, previous to the using "of the sulphate, was contracted, thickened and indurated, so "that it was quite difficult to introduce a finger, but after the "use of the remedy the induration and thickening disappeared, "so that two fingers can be inserted, and the opening seems "thin and quite dilatable. I believe this action has extended "over the whole abscess wall, with the exfoliation of the pyo-"genic membrane, which came away in shreds and patches "(much to my surprise). The only inconvenience was that "there was somewhat of a seeping of the sulphate into the

"rectum, which resulted in some irritation. Mrs. T. is im-
"proving, and feeling stronger and greatly encouraged."

The abscess healed rapidly after this. Her pulse became normal, and her appetite unusually keen. By the first of September, two weeks after the cauterization, she had gained thirteen pounds, declared herself to be as well and strong as ever in her life, and was visiting all over the town in almost childish enjoyment of her regained health and vigor. A shallow depression was all that was left of the abscess. The menstrual flow had not shown itself since the commencement of her illness.

Early in September she was attacked with the then prevalent epidemic, dysentery, and died on the 23rd instant.

At the post-mortem examination, made about thirty hours after death, I was somewhat hampered on account of a promise, exacted by the husband, that no organ should be taken out of the body, and by the fact that I had but thirty minutes for work before train-time. The body had again become extremely emaciated. Abdomen was flat. An incision was made from a little above the umbilicus to the pubic bone. The pelvis was filled posteriorly with a solid mass of plastic tissue, which had drawn the uterus backwards to within about half an inch of the sacrum, so as to put the anterior vaginal wall upon the stretch, and had buried the uterus and other pelvic organs in its substance. Both round ligaments were seen issuing from this mass. It was necessary to cut down about half an inch before reaching the depressed uterus, and to tear through solid tissue behind it to arrive at the rectum below. The finger broke through into the rectum, behind the dimpled cicatrix that marked the site of the former outlet of the abscess. The left broad ligament was then felt to be represented by, or inclosed in, a tough band half an inch thick antero-posteriorly, extending from the uterus to the left side of the pelvis. The left ovary could not be found. A small flat piece of what seemed to be ovarian tissue was found adhered to the bladder on the right side. The right broad ligament was apparently disorganized and inseparable from the plastic deposit. The rectum was held inflated at the point where it issued from the pelvis, was dark colored and injected on its external surface, and blackish and softened on the internal. Neither the appearance nor the odor of an abscess could anywhere be discovered.

Allow me in this connection to quote the following extracts from a letter, written to me by Dr. Marr on the twenty-eighth day of the same month:

"Yours of the 26th is at hand. * * * Owing to the "hurried examination, I extended it somewhat after you left, "with the result of fully confirming your views of the uterus "and its appendages. * * * I extended the examination "to the alimentary canal, and found the conditions peculiar to "that disease (epidemic dysentery) extending over the whole "of the large intestine and a part of the smaller, with intense "congestion of the stomach and peritoneum. The mucous "membrane of the larger bowel was very dark, ulcerated "and sloughy, the inflammation extending to the muscular "coat."

There seem to have been two hinges, as it were, upon which the treatment of this abscess turned: First, the operation per rectum; second, the cauterization by sulphate of copper. Both secured a large opening at the lowest portion of the pyogenic cavity, and brought away the unhealthy granulation-tissue. Had the patient consented to have the unobstructed outflow of the pus maintained by one or two subsequent dilations, similar to the first one, the cure would undoubtedly have been more rapid. As it was, the contracting sphincter and abscess outlet rendered the drainage and irrigation imperfect. Progress toward recovery was, however, again inaugurated upon the melting away, by the sulphate of copper, of the newly and imperfectly formed cicatricial tissue, reproducing the opening made at the time of the operation; and by the destruction of the degenerative deposits, and cauterization of the chronic pyogenic surface. The only kind of treatment preferable to this free drainage and clearing out method, is the strictly antiseptic, which, after the pus has once found a way into the rectum, can only be accomplished by first closing this septic inlet.

The treatment by a counter opening in the vagina is much less preferable, because a recto-vaginal fistula, difficult of cure, and liable, like anal fistula, to inoculate the system with tuberculosis, would be left.

The treatment by abdominal incision cannot for a moment be entertained, for at least two reasons:

1st. It is necessarily followed by a recto-abdominal fistula of great length, which is incapable of being promptly cured, and is apt to become an unfailing source of systemic infection. Those patients already operated upon, as far as reported, have usually either died shortly, or within a year or two, imperfectly cured. They would have, on an average, lived about as long without the operation. In fact, it is not impossible that one such, whom I had, previous to the operation, an opportunity of watching for a short time, would finally have recovered through the process of nature. To operate as does Lawson Tait, before the abscess has discharged, and then treat it antiseptically through its single opening, is an entirely different matter.

2nd. The danger of an abdominal incision should never be incurred without a prospect of compensation in the way of bettering the patient's chances of recovery. Neither theory nor practice as yet proves such compensation to be attainable.

In some cases one dilatation per rectum, without after-treatment, has sufficed for a cure; in other cases two or more, with subsequent antiseptic irrigations, have become necessary. But as a general rule it may be said that, unless instituted too late, the procedure is safe and the recovery sure.

3100 Forest Avenue.

